# Supplementary figures and images for: Dendrobine Alleviates Cellular Senescence and Osteoarthritis via the ROS/NF-κB Axis
Source: Int J Mol Sci. 2023 Jan 25;24(3):2365. doi: 10.3390/ijms24032365 (PMC9916903; doi:10.3390/ijms24032365)

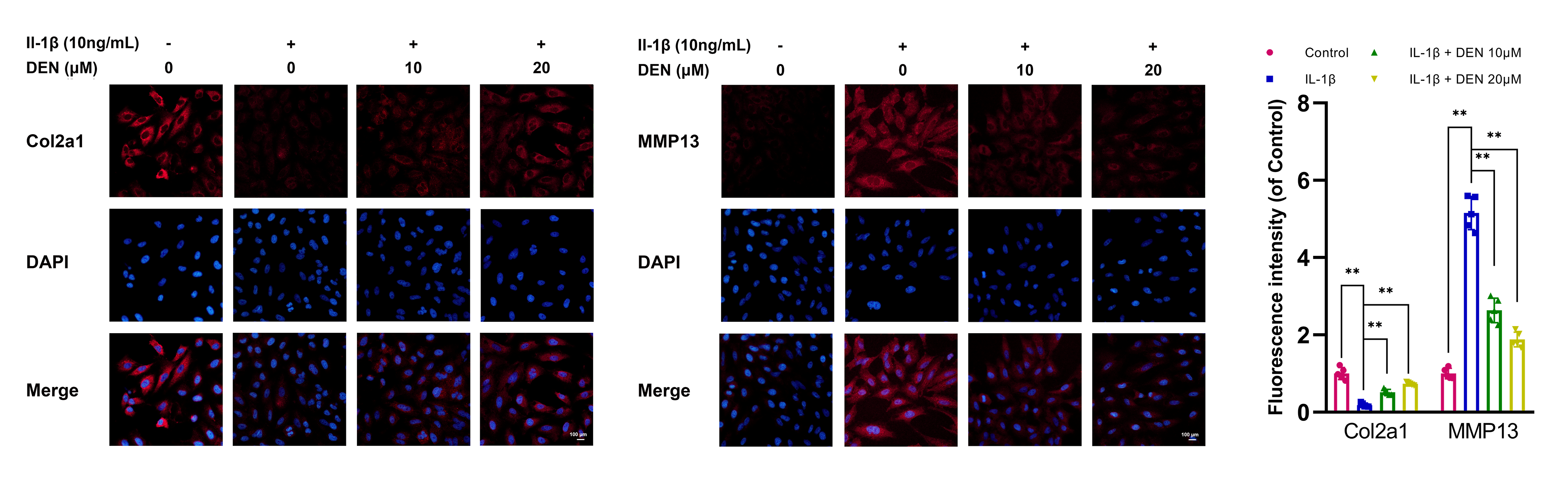

Supplement: Supplementary file 1 [file ijms-24-02365-s001.zip › Supplementary Files/Figure S1.tif]

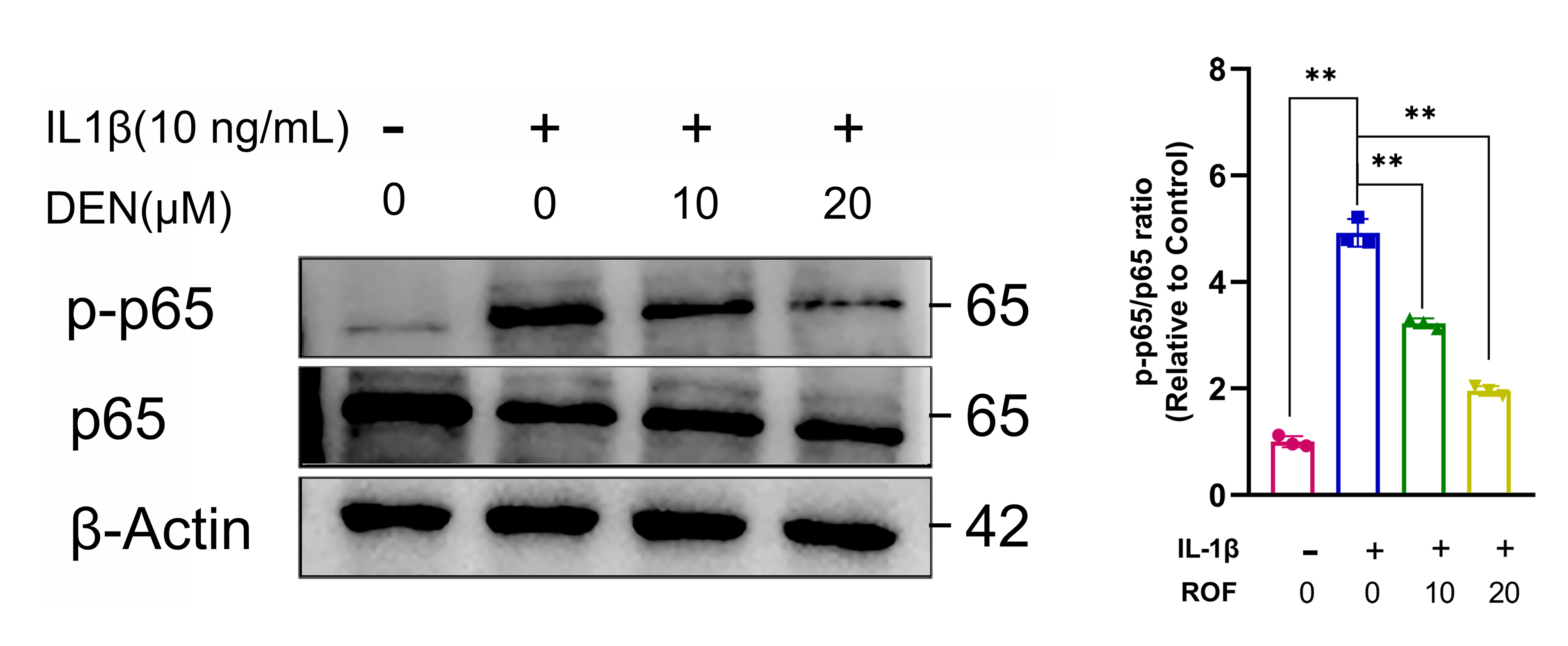

Supplement: Supplementary file 1 [file ijms-24-02365-s001.zip › Supplementary Files/Figure S2.tif]

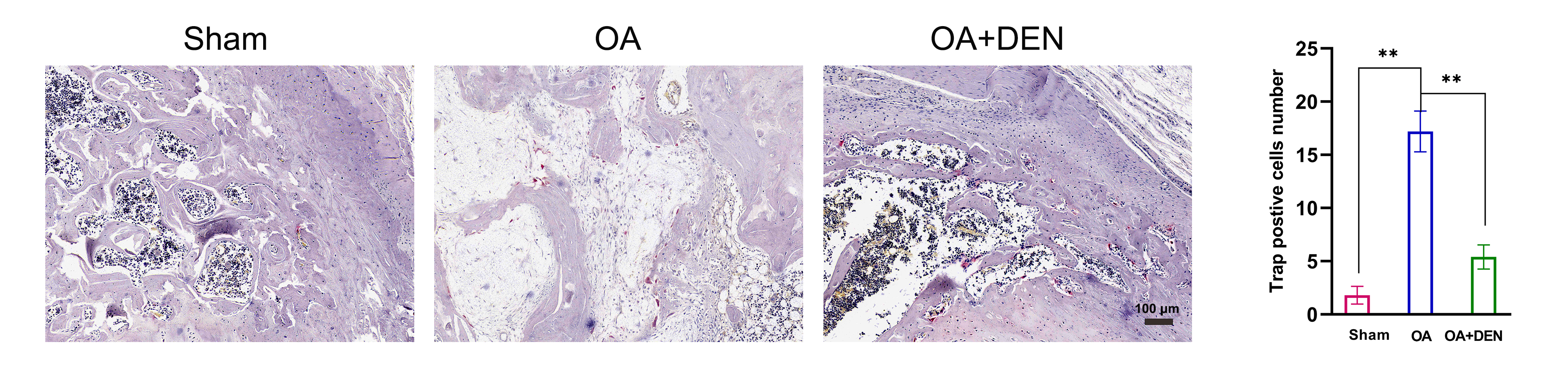

Supplement: Supplementary file 1 [file ijms-24-02365-s001.zip › Supplementary Files/Figure S3.tif]
